# Zoonotic Visceral Leishmaniasis: New Insights on Innate Immune Response by Blood Macrophages and Liver Kupffer Cells to *Leishmania infantum* Parasites

**DOI:** 10.3390/biology11010100

**Published:** 2022-01-09

**Authors:** Armanda Viana Rodrigues, Ana Valério-Bolas, Graça Alexandre-Pires, Maria Aires Pereira, Telmo Nunes, Dário Ligeiro, Isabel Pereira da Fonseca, Gabriela Santos-Gomes

**Affiliations:** 1 Global Health and Tropical Medicine, GHTM, Instituto de Higiene e Medicina Tropical, IHMT, Universidade Nova de Lisboa, UNL, Rua da Junqueira 100, 1349-008 Lisboa, Portugal; armanda.rodrigues@ihmt.unl.pt (A.V.R.); ana.bolas@ihmt.unl.pt (A.V.-B.); mapereira@esav.ipv.pt (M.A.P.); 2 CIISA, Centre for Interdisciplinary Research in Animal Health, Faculty of Veterinary Medicine, University of Lisbon, Av. Universidade Técnica, 1300-477 Lisbon, Portugal; gpires@fmv.ulisboa.pt (G.A.-P.); ifonseca@fmv.ulisboa.pt (I.P.d.F.); 3 Polytechnic Institute of Viseu, Agrarian School, Quinta da Alagoa-Estrada de Nelas Ranhados, 3500-606 Viseu, Portugal; 4 Microscopy Center, Faculty of Sciences, Campo Grande, 1749-016 Lisboa, Portugal; telmonunes@hotmail.com; 5 IPST-Centro de Sangue e Transplantação de Lisboa, Alameda das Linhas de Torres, 117, 1749-005 Lisbon, Portugal; dario@ipst.min-saude.pt

**Keywords:** blood macrophages, Kupffer cells, innate immunity, *Leishmania infantum*, zoonotic visceral leishmaniasis

## Abstract

**Simple Summary:**

*Leishmania infantum* is a parasite that causes zoonotic visceral leishmaniasis, a disease that affects humans, wild and domestic animals, mainly domestic dogs. This parasite develops inside the macrophages and, concealed inside these cells, the parasite can invade the inner organs such as the spleen and liver, causing life-threatening disease. Therefore, a better understanding of the immune mechanisms exhibited by the macrophages when facing this parasite is needed to improve control strategies. Macrophages are cells of the immune system, existing in the peripheral blood and associated with different tissues in the mammal body, having the task to protect against microbiological threats. Interestingly, *Leishmania* can manipulate the macrophages into a non-active ghost-like state, allowing the parasite to stay in the host. The liver, which is a vital organ and a target for the parasite, has a resident population of macrophages designated as Kupfer cells. Thus, this study aims to evaluate the innate immune response exhibited by these two distinct cell populations—blood-macrophages and Kupfer cells—against *Leishmania* parasites. Our findings showed that *Leishmania* takes advantage of the natural disposition of blood macrophages to perform phagocytosis and facilitate parasite internalization, which rapidly subverts the immune mechanisms of macrophages. On the other hand, Kupfer cells are not extensively immune activated in the first contact with the parasite but seem to be more efficient in parasite infection, thus contributing to the ability of the liver to naturally restrain *Leishmania* dissemination.

**Abstract:**

*L. infantum* is the aetiological agent of zoonotic visceral leishmaniasis (ZVL), a disease that affects humans and dogs. *Leishmania* parasites are well adapted to aggressive conditions inside the phagolysosome and can control the immune activation of macrophages (MØs). Although MØs are highly active phagocytic cells with the capacity to destroy pathogens, they additionally comprise the host cells for *Leishmania* infection, replication, and stable establishment in the mammal host. The present study compares, for the first time, the innate immune response to *L. infantum* infection of two different macrophage lineages: the blood macrophages and the liver macrophages (Kupffer cells, KC). Our findings showed that *L. infantum* takes advantage of the natural predisposition of blood-MØs to phagocyte pathogens. However, parasites rapidly subvert the mechanisms of MØs immune activation. On the other hand, KCs, which are primed for immune tolerance, are not extensively activated and can overcome the dormancy induced by the parasite, exhibiting a selection of immune mechanisms, such as extracellular trap formation. Altogether, KCs reveal a different pattern of response in contrast with blood-MØs when confronting *L. infantum* parasites. In addition, KCs response appears to be more efficient in managing parasite infection, thus contributing to the ability of the liver to naturally restrain *Leishmania* dissemination.

## 1. Introduction

Monocytes are a pleiotropic leucocyte population derived from the bone marrow that constitute part of the mononuclear phagocyte system (MPS). These cells circulate in the bloodstream and perform crucial functions as effector cells of the innate immune system. They express several chemokine receptors and adhesion molecules and are easily engaged to sites of infection [1]. Blood circulating monocytes can be activated and terminally differentiated into macrophages (MØs), constituting a specialized cell population able to perform extensive phagocytosis of invading microorganisms and foreign bodies, and to clearance of cell debris, modified or damaged cells, that do not express specific markers of normal cells [2]. Monocytes/macrophages, as well as polymorphonuclear neutrophils (PMNs), express several pattern recognition receptors (PRRs), such as Toll-like receptors (TLR) and nucleotide-binding oligomerization domain-like (NOD) receptors. These innate immune receptors are capable of sensing highly conserved and distinct pathogen-associated molecular patterns (PAMPs) as well as damage-associated molecular patterns (DAMPs), produced by body cells in the event of cellular and/or tissue injury [3,4]. TLRs constitute transmembrane complex proteins that can sense PAMPs [such as lipids, nucleic acids and lipopolysaccharide (LPS)] in the extracellular and/or intracellular space [3]. NODs function exclusively as intracytoplasmic sensors and can recognize different structural core motifs, for example, peptidoglycan, a component of bacterial cell walls that is recognized by NOD1 and NOD2 [5].

Mammals have several specialized MØs populations, usually associated with tissues, that may differ from blood-MØs in lineage origin as well as in immunological behavior. These include the Kupffer cells (KCs) in the liver, red pulp macrophages in the spleen, Langerhans cells in the skin, cardiac-resident MØs, and alveolar MØs in the lung. Tissue MØs act as sentinels, detecting pathogens and cellular injuries, and constitute themselves a different cell subset [6,7]. Only recently has the origin of this tissue-macrophage population, such KCs, been established. They are originated from the fetal yolk-sack, and established during embryonic development, persisting independently of blood monocytes as a predominantly self-renew population [6,8].

KCs constitute the largest population of resident tissue MØs in the mammal body and these cells undertake crucial innate immune functions. KCs are mainly localized in the hepatic sinusoids, where, from this prime location, can proficiently perform immune surveillance on pathogens, particulates and immunoreactive material derived from the gastrointestinal tract and entering the liver via the portal vein or arterial circulation, working as the first line of defense [9]. Although KCs are capable of being immunologically activated, in a healthy liver, KCs exhibit a dominant immuno-tolerant phenotype, primed by the natural liver’s tolerogenic microenvironment. This tolerance is essential to prevent undesired immune responses in the face of incoming immunoreactive materials from the bloodstream into the liver. However, under certain conditions, KCs can be activated and shift from tolerogenic phenotype to a pathologically activated state that may result in hepatocellular injury and damage if not properly controlled [10]. Thus, preserving a balanced functional activity of KCs is critical for the maintenance of a healthy organism.

Therefore, MØs and KCs can be effective phagocytic cells with the ability to inactivate pathogens. On the other hand, these cells comprise the host cells for *Leishmania* infection, replication and stable dispersion inside the mammal host. *Leishmania* parasites are highly adapted to the hostile environment inside the phagolysosome, being even able to manipulate MØs immune activation [11].

*L. infantum* comprises the aetiological agent of zoonotic visceral leishmaniasis (ZVL) and canine leishmaniasis (CanL). In a natural infection, *L. infantum* promastigotes are inoculated into the mammal dermis by the sandfly during a blood meal. Parasites are then phagocyted by PMNs and blood macrophages [12]. *Leishmania* parasites have evolved several mechanisms to avoid and subvert macrophage activity, evading the fusion of phagosome with the lysosome and differentiating into the intra-macrophagic form, the amastigote [13,14]. Once inside the circulating blood-MØs parasites are transported into the inner organs, namely to the spleen and liver. In the liver, amastigotes encounter KCs, which can be infected and where the parasite can persist. Thus, MØs play a dual role in *Leishmania* infection as these cells can promote the destruction of internalized parasites and also provide a safe place for *Leishmania* replication and dispersion to MPS. MØs are key to disease progression and the success or failure of the infection depends on the interplay between the parasite and the host’s immune response.

The present work aimed to contribute with new insights on the close evolution of *L. infantum* and the dog macrophage, the parasite-host cell. The present study compares, for the first time, the innate immune response of two different macrophage lineages in response to *L. infantum* infection. Our findings showed that *L. infantum* can take advantage of the natural predisposition of blood-MØs to phagocyte pathogens to survive, rapidly subverting the activation cell’s immune mechanisms into a dormancy state, by decreasing innate pattern recognition receptors (PRRs) and cytokine expression, and at the same time extending host cell viability, buying time to establish a visceral infection in the host. On the other hand, KCs, which are primed, since origin, for immune tolerance, are not extensively activated in the presence of *L. infantum*, presenting residual levels of PPRs and cytokine generation. Interestingly, KCs can overcome the parasite’s induced dormancy, exhibiting a selection of immune mechanisms, such as extracellular trap formation (METs) and nitric oxide production. Altogether, KCs reveal a different pattern of response in contrast to blood-MØs when facing *L. infantum* parasites and appear to be more efficient in managing parasite infection, thus contributing to the ability of the liver to naturally restrain *Leishmania* dissemination.

## 2. Materials and Methods

### 2.1. Kupffer Cell Purification and Culture

Canine KCs were isolated from a liver lobule after tissue disruption and application of a double Percoll^®^ gradient. The protocol is described by Rodrigues and colleagues [15]. Briefly, a liver lobule was externally cleaned with sterile 0.9% NaCl and sliced into Gey’s balanced salt solution (Sigma Aldrich, Saint Louis, MO, USA) supplemented with 0.2% (*m*/*v*) pronase (Sigma-Aldrich) and 0.8 µg.mL^−1^ DNAse (Roche, Basel, Switzerland). The mixture was left in agitation (800 rpm) for 1 h at 37 °C in a humidified atmosphere with 5% CO_2_. After cleaning the suspension, single cells suspension (10 mL) was overlaid into a double Percoll^®^ (GE Healthcare Bio-Sciences AB, Uppsala, Sweden) gradient formed by two successive layers of 25% and 50% Percoll. After centrifugation, the intermediate fraction was collected, washed with PBS 1×, and resuspended in RPMI medium (BioWhittaker Lonza, Basel, Switzerland) supplemented with 10% (*v*/*v*) heat-inactivated fetal bovine serum (FBS, Sigma-Aldrich) L-Glutamine 0.2 mM (Merck, KGa, Darmstadt, Germany), 100 U.m^−1^ of penicillin and 100 µg.mL^−1^ of streptomycin (Sigma-Aldrich). Isolated cells had approximately 95% of viability (determined by trypan blue exclusion dye in a Neubauer’s chamber). Isolated cells were cultured for 7 days in a six-well plate (VWR) with supplemented RPMI and macrophage colony-stimulating factor (10% *v*/*v* M-CSF) to ensure KCs maximum cell differentiation, at 37 °C in a humidified atmosphere with 5% CO_2_. and routinely observed under an inverted microscope (Olympus, CKX41, Olympus Corporation, Tokyo, Japan). Cell images were acquired with an Olympus CS30 camera.

### 2.2. Separation of Peripheral Blood Monocytes and Differentiation to Macrophages

Canine peripheral blood was collected to a tube with anticoagulant Citrate Phosphate Dextrose Adenine Solution (CPDA) and utilized for the isolation of blood monocytes and for testing for *L. infantum*, *Dirofilaria immitis*, *Babesia*/*Theleria* spp., *Ehrlichia*/*Anaplasma* spp., *Mycoplasma haemocanis* and *Ricketsia*. Only animals that tested negative for all the pathogens were included in final results to avoid any bias. The procedure for the isolation of blood monocytes. The procedure is described by Pereira and colleagues [12] and briefly comprises the overlaying 2 mL of peripheral blood on a double gradient of Hystopaque^®^ density 1119 (Sigma-Aldrich) and Hystopaque^®^ density 1077 (Sigma-Aldrich) and centrifugation. The cell ring, enriched in monocytes, at the interface of the two gradients was collected and washed. Red blood cells were lysed and cells viability (about 95%) and concentration were determined by trypan blue exclusion dye in a Neubauer’s chamber. Monocyte differentiation into MØs was induced by culture isolated cells in supplemented RPMI medium with 10% (*v*/*v*) of M-CSF for four days. Flow cytometry immunophenotype of the isolated cells using universal macrophages markers (CD14, CD11c, CD1a) was performed to ensure that the isolated cells were in fact macrophages (see Figure S1). The vast majority of the isolated cells express these markers, confirming their identity as macrophages.

### 2.3. L. infantum Parasites

*L. infantum* (MHOM/PT/89/IMT151) parasites were maintained at 24 °C in Schneider medium with L-glutamine (SCHN, Sigma-Aldrich) supplemented with 10% (*v*/*v*) of heat-inactivated FBS (Sigma-Aldrich) and penicillin-streptomycin (Biochrom, GmbH, Irvine, UK) at 100 U/mL and 100 μg.mL^−1^ (complete SCHN medium). Only virulent parasites with less than five passages were used [16]. *L. infantum* amastigotes differentiation in axenic conditions was performed using stationary phase promastigotes (2 × 10^6^ parasites.mL^−1^) inoculated into pH 5.5 complete SCHN medium supplemented with 2% of filtered human urine (from a healthy male donor) [15]. Cultures were incubated at 37 °C, in a humidified atmosphere with 5% CO_2_ for two to three weeks to allow complete amastigote transformation. The amastigote differentiation was followed by inverted optical microscopy and confirmed morphologically by scanning electronic microscopy (JEOL5200-LV, JSM Electron Microscopes, Tokyo, Japan). Green fluorescent protein (GFP)-expressing *L. infantum* promastigotes were used to generate GFP-amastigotes [17]. Similar culture conditions were used and SCHN medium was supplemented with 25 μg.mL^−1^ of geneticin (Sigma-Aldrich). Amastigote differentiation was confirmed under a fluorescent microscope equipped with a GFP filter.

### 2.4. L. infantum Infection of Macrophage and Kupffer Cells

For infection of blood-MØs was used a proportion of three *L. infantum* virulent promastigotes per cell. To mimic the natural in vivo infection progression, KCs were exposed to axenic *L. infantum* amastigotes at a proportion of three parasites per cell. Blood-MØs and KCs exposed to parasites were maintained in supplemented RPMI medium and incubated at 37 °C in a humidified atmosphere with 5% CO_2_. Cells and supernatants were collected at 1.5 h, 3 h and 5 h post-infection for further analysis. As a positive inducer of inflammation, Escherichia coli lipopolysaccharide (LPS, 1 µg.mL^−^^1^) (Sigma-Aldrich) was added up to cells and samples were collected from duplicated wells at 1.5 h, 3 h, and 5 h of incubation with LPS. To ensure that infected blood-MØs remain viable after 5 h post-infection with *L. infantum* virulent promastigotes, a cell viability assay using Annexin V and propidium iodide (Invitrogen, Paisley, Scotland) was performed by flow cytometry (Cytoflex, Beckman Coulter, Brea, CA, USA).

### 2.5. Scanning Electron Microscopy (SEM)

KCs, as well as axenic *L. infantum* amastigotes and promastigotes, were prepared for SEM observation by washing with PBS and followed by fixation with 2% paraformaldehyde (*m*/*v*) in PBS and posterior washing with ice-cold PBS. Blood-MØ were fixed as described in Pereira and colleagues [12]. For further processing, cells were dehydrated in a graded ethanol series [30, 50, 70, 80, 90, and 100% (*v*/*v*)] and dried using the critical point drying method, coated with gold-palladium and mounted on stubs. Preparations were observed in a scanning electronic microscope (JEOL5200-LV) and digital images acquired.

### 2.6. Evaluation of Cell-Parasite Interaction by Microscopy

Assessment of cell-parasite interaction was performed on KCs exposed to axenic amastigotes and GFP-amastigotes as well as in blood-MØs exposed to promastigotes, using different techniques of microscopy. Fluorescent microscopy as described by Rodrigues and colleagues [15] was used to analyze GFP-amastigotes impact on macrophage markers CD68 and lysozyme. Briefly, cells were washed and fixed with 2% paraformaldehyde in PBS and permeabilized overnight with PBS 1% (*v*/*v*) Tween-20 (Sigma-Aldrich) and 0.2% (*v*/*v*) FBS. Antibodies (anti-CD68, polyclonal FITC conjugated sc-7083-Santa-Cruz dilution 1:250; anti-lysozyme, primary rabbit polyclonal antibody, ab74666-Abcam, dilution 1:10 and secondary goat anti-rabbit polyclonal Alexa fluor 647, ab150091-Abcam, dilution 1:1000) in PBS 0.125% (*v*/*v*) FBS and 1% (*v*/*v*) Tween-20 buffer were employed. TO-PRO^®^-3 antifade mounting medium (Thermo Fisher Scientific, Waltham, MA, USA) was used and images were acquired with a Leica TCS SP2 Laser Scanning Confocal Microscope (Leica Microsystems GmbH, Wetzlar, Germany). KC incubated with non-GFP amastigotes were washed and fixed and stained for nucleic acids with DAPI (Thermo Fisher Scientific) and images were acquired with an Eclipse 80i INTENSILIGHT C-HGFI Epi-fluorescence microscope and NIS-Elements software from Nikon (Nikon Instruments Inc., Melville, NY, USA). To verify blood-MØs infection, cells were detached with PBS-EDTA (1×) and a cytospin (StatSpin CytoFuge2, IRIS International Inc., Chatsworth, Los Angeles, CA, USA) was performed. Cells were fixed with methanol, stained with a Hemacolor staining kit (Merck Millipore, KGaA, Darmstadt, Germany) and observed by optical microscopy (Olympus BX51, Olympus Corporation, Tokyo, Japan) for internalized parasites.

### 2.7. Nitrite Detection

To quantify the nitrate/nitrite concentration in cellular supernatants, these were collected, centrifuged to remove cell debris and stored at −20 °C until further analysis. A commercial kit Nitrate/Nitrite Colorimetric Assay kit (Abnova, Taipei City, Taiwan) was performed accordingly to the manufacture’s recommendations. Final results were expressed as µM of nitrate + nitrite and a ratio to control (non-infected cells) was calculated.

### 2.8. Real Time-PCR Analysis

To analyze the innate immune response and cytokine generated by blood-MØs and liver KCs in response to *L. infantum* parasites, real-time PCR was performed. Primers used in the present work are described by Rodrigues and colleagues [15] and were selected using data from published works or were designed using Primer-BLAST software. Cellular RNA extraction was executed, according to the manufacturer’s recommendations (NZY Total RNA Isolation kit, Nzytech genes & enzymes, Lisboa, Portugal) and processed into cDNA using NZY First-strand cDNA Synthesis Kit (Nzytech genes & enzymes). Real-time polymerase chain reaction (PCR) quantitative method was performed in 7500 FAST Real-Time PCR System thermal cycler (Applied Biosystems, Foster City, CA, USA). A total volume of 20 µL, containing 2 µL of cDNA, 10 µL of SensiFAST SYBR Lo-ROX (Bioline, Reagents Ltd., Meridian Bioscience, London, UK) and primers (Stabvida, Caparica, Portugal) (20 pmol.µL^−1^) was used for the PCR run (5 min at 95 °C, followed by a total of 40 cycles of 30 s at 95 °C, 30 s at primer annealing temperature, and melting curve. External cDNA standards were constructed for all target genes by cloning PCR fragments into a pGEM^®^-TEasy Vector according to the manufacturer’s recommendations (Promega, Madison, WI, USA) and as described by Rodrigues and colleagues [18]. The concentration of cDNA standards was determined by measuring the optical density (OD) at 260 nm and serial dilutions were used as standard curves. The number of copies of each gene and sample was normalized to the housekeeping gene β-actin and results were expressed as the number of copies per 1000 copies of β-actin.

### 2.9. Statistical Analysis

The non-parametric Wilcoxon test for two related samples was used to compare differences between time points and different experimental conditions for blood-MØs or liver KCs. Data analysis was performed using the GraphPad Prism 8 software (San Diego, CA, USA). Samples from 10 (*n* = 10) dogs were used in the present study. All samples were evaluated in triplicate (minimum). Figures presenting images of cells using several microscopy techniques represent the best obtained results for all the isolated cell samples combined (*n* = 10). A significance level of 5% (*p* < 0.05) was used as indicative of statistical significance. The present study evidenced several outlying values that may reflect canine natural population diversity; as for the present study no restrictions on breed, gender or age were applied.

### 2.10. Ethical Considerations

The cells used in the present study were obtained from dogs euthanized by the competent authorities, and no live animals were used. The authors had no intervention in animal culling.

## 3. Results

### 3.1. Axenic L. infantum Amastigotes Can Infect KCs

**Figure 1:**
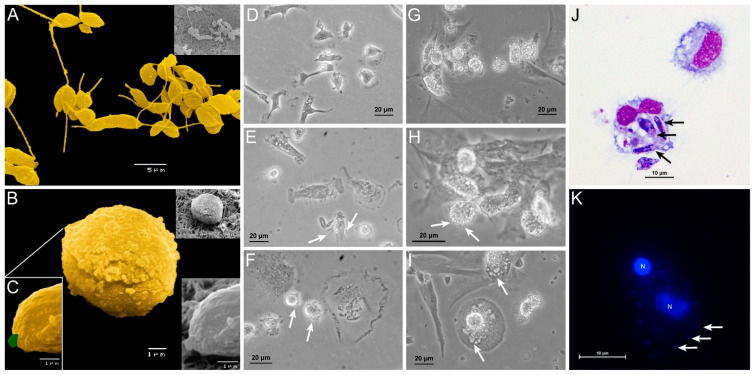
Canine blood-MØs and KCs exposed to *L. infantum* virulent promastigotes or axenic amastigotes. *L. infantum* virulent promastigotes (yellow) showing elongated flagellum characteristic of *L. infantum* metacyclic promastigotes (**A**) and axenic amastigotes (**B**) with a characteristic oval body (yellow) and residual flagella (**C**) (green) were observed under SEM. The acquired images were artificially colored. Blood-MØs (uninfected-**D**) were exposed to virulent promastigotes for 3 h (**E**) and 5 h (**F**). Interaction of promastigotes with the MØ membrane can be observed (white arrows) along with parasite internalization (**E**,**F**). Uninfected KCs (**G**) and KCs exposed to axenic *L. infantum* amastigotes for 1.5 h (**H**) and 5 h (**I**). Internalization of amastigotes by KCs (white arrows) can be observed (**I**). Blood-MØs exposed to virulent *L. infantum* promastigotes for 3 h stained with Hemacolor staining kit were observed by optical microscopy (magnification 1000×) (**J**). Black arrows indicate promastigote internalization. KCs exposed to *L. infantum* amastigotes for 3 h and stained with DAPI (**K**) (blue) were observed by fluorescence microscopy. Acquired images show parasites binding to the KCs membrane. N-KCs nucleus.

In the present study, blood-MØs and KCs were infected with different *L. infantum* evolutive morphological forms, mimicking the natural progression of parasite infection. The immune activation evidenced by circulating blood-MØs infected by *L. infantum* promastigotes, the parasite morphological form deposit by sand fly in the dermis of the host, had been compared with KCs exposed to *L. infantum* amastigotes, the parasite form that reaches the internal organs of mammal host. Virulent *L. infantum* promastigotes (less than three culture passages) were used (Figure 1A) to differentiate amastigotes. In axenic conditions, parasites acquire an amastigote-like morphology (Figure 1B), with an oval shape and a residual flagellum visible in the flagellar pocket (Figure 1C).

Canine blood-MØs were exposed to *L. infantum* virulent promastigotes and parasite internalization was observed (Figure 1D–F,J). After 3 h of incubation, promastigotes were internalized by blood-MØs and more than one parasite per cell could be observed (Figure 1J). In turn, KCs were exposed to amastigotes (Figure 1G–I,K). After 5 h of incubation, *L. infantum* axenic amastigotes were internalized by KCs (Figure 1I,K), while clearance of extracellular amastigotes was noticed (Figure 1H,I). Amastigote internalization by KCs was observed by fluorescence microscopy (Figure 1K). No evidence of KCs or blood-MØs lysis was detected during the period of infection.

**Figure 2:**
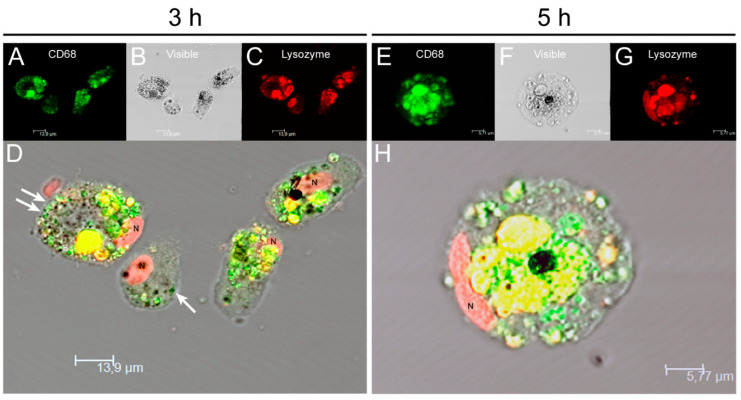
Internalization of *L. infantum* parasites by canine KCs. After 3 h (**A**–**D**) and 5 h (**E**–**H**) of exposure to *L. infantum* GFP amastigotes (white arrows), KCs were stained for CD68 (green), lysozyme (red) and nucleus (N) and fluorescent and visible light images were acquired. Co-localization of CD68 and lysozyme in lysosomal compartments (yellow) can be observed in overlapping images (**D**,**H**). CD68 and lysozyme accumulation can be noticed at 5 h of parasite exposure (**H**).

**Figure 3:**
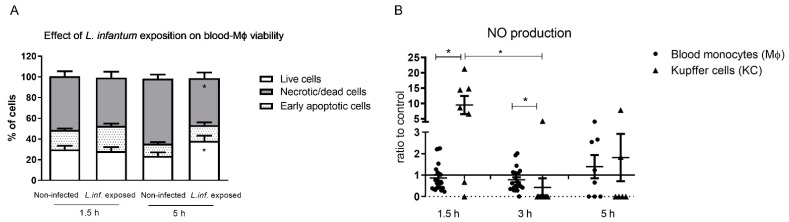
Viability of canine blood-MØs and nitric oxide (NO) production by KCs and blood-MØs exposed to *L. infantum*. The viability of blood-MØs was assessed by flow cytometry with annexin-V and propidium iodide staining (**A**). Data are represented by stacked bars showing mean and standard deviation (*n* = 6). Results of NO production by blood-MØs exposed to virulent promastigotes and KCs exposed to axenic amastigotes were normalized to non-infected cells (**B)**. Individual values are indicated as black dots (blood-MØs) and black triangles (KCs) with mean and standard deviation (*n* = 10). Non-parametric Wilcoxon test was used for statistical comparisons. * (*p* < 0.05) indicate significant differences.

**Figure 4:**
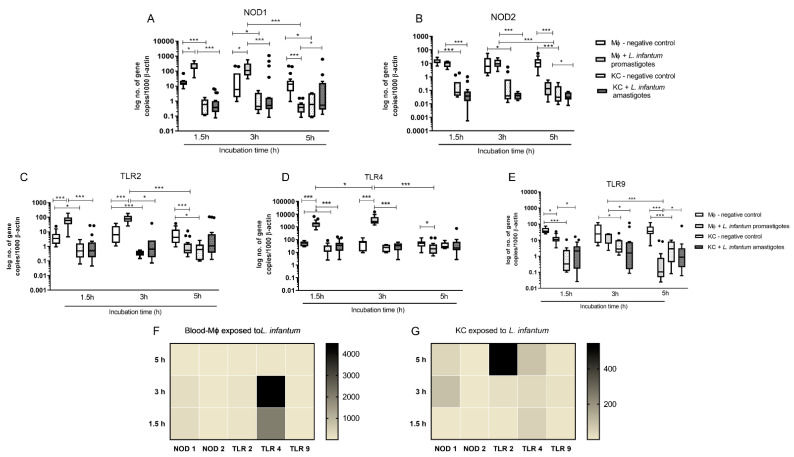
Gene expression of innate immune receptors in *L. infantum* infected KCs and blood-MØ. Blood-MØs were exposed to promastigotes and KCs to amastigotes. The number of copies of NOD1 (**A**), NOD2 (**B**), TLR2 (**C**), TLR4 (**D**), and TLR9 (**E**) mRNA was determined by real-time PCR at 1.5 h, 3 h, and 5 h of parasite exposure. Results are expressed by whisker box-plot (Tukey). Outlier values are indicated by black dots. The non-parametric Wilcoxon test was used for statistical comparisons (*n* = 10). * (*p* < 0.05) and *** (*p* < 0.0001) indicate significant differences. Heat maps represent the mRNA accumulation of innate sensors by blood-MØs (**F**) and KCs (**G**) during parasite exposure.

**Figure 5:**
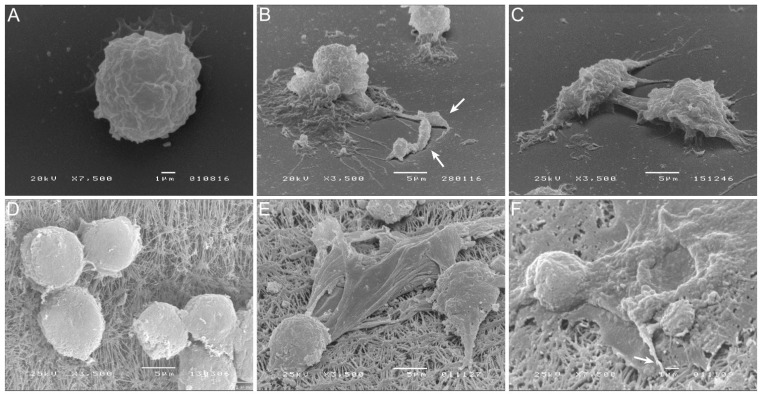
Blood-MØs phagocyting promastigotes and KCs emitting extracellular traps (ETs) in response to *L. infantum*. Uninfected blood-MØs (**A**) and blood-MØs exposed to virulent *L. infantum* promastigotes for 3 h (**B**,**C**) were observed by SEM and images were acquired. White arrows point out fiber-like structures entrapping promastigotes. Uninfected KCs (**D**) and KCs releasing ETs after 5 h of incubation with *L. infantum* axenic amastigotes (**E**,**F**) can be observed. White arrows indicate a fiber-like structure and cytoplasm extravasation.

To analyze in more detail the effect of internalization of amastigotes by KCs, cell staining for CD68 and lysozyme (macrophage markers) were performed and fluorescence microscopy images were acquired (Figure 2). Following previous descriptions, CD68 is a promoter of phagocytosis, and lysozyme is an enzyme that hydrolysis bacterial peptidoglycans. Both components were found co-localized in cytoplastic granules. It was observed an increase in CD68 and lysozyme expression from 3 h (Figure 2D) to 5 h (Figure 2H) after amastigote exposure. GFP-amastigotes internalized by KCs were observed in a peripheral position (Figure 2D).

### 3.2. L. infantum Infected Blood-MØs Have Increased Lifespan

Blood-MØs viability was assessed to ensure that cells remain functional after infection with *L. infantum* (Figure 3A). Remarkably, the exposition of blood-MØs to parasites resulted in cell’s increasing their lifespan (P _5 h_ = 0.0137) and presented less necrotic/dead cells (P _5 h_ = 0.0068) when compared to non-infected cells. Non-infected blood-MØs viability remained similar between 1.5 h and 5 h of culture, reflecting the natural cell turnover in the population.

Nitric oxide (NO) production of blood-MØs and KCs were also assessed, as a measure of cell activation, since NO is a potent leishmanicidal molecule produced by activated macrophages. Both blood-MØs and KCs were able to activate NO production when exposed to *L. infantum* (Figure 3B). However, infected blood-MØs exhibited a constant but low NO production throughout the incubation time. KCs exposed to amastigotes, at early incubation time, produced higher levels of NO (P _1.5 h_ = 0.0178) when compared to blood-MØs exposed to promastigotes. After 3 h of parasite exposure, KCs reduced NO levels (P _3 h_ = 0.0313) and infected blood-MØs exhibited a higher NO production (P _3 h_ = 0.0004). After 5 h of parasite exposure, KCs evidencing a slightly higher NO level than blood-MØs, although not statistically significant.

### 3.3. Infected Blood-MØs Display a Peak of PRR Gene Expression in Response to L. infantum

Innate immune receptors for intracellular and cytoplasmatic PAMPs (NOD1, NOD2 and TLR9) and extracellular PAMPs (TLR2 and TLR4) were accessed. Overall, blood-MØs exposed to *L. infantum* promastigotes revealed higher levels of induction of gene expression for all tested receptors, as well as a different activation pattern from KCs (Figure 4).

Non-infected blood-MØs naturally exhibited higher levels of NOD1 (P _1.5 h_ < 0.0001, P _3 h_ = 0.0089, P _5 h_ = 0.0014) and NOD2 mRNA (P _1.5 h_ < 0.0001, P _3 h_ = 0.0007, P _5 h_ < 0.0001) when compared with resting KCs (Figure 4A,B). After 1.5 h and 3 h of exposure to *L. infantum* promastigotes, blood-MØs showed a significantly higher accumulation of NOD1 mRNA (P _1.5 h_ = 0.0001, P _3 h_ = 0.0009). Canine KCs exposed to *L. infantum* amastigotes did not exhibit increased NOD1 nor NOD2 gene expression, maintaining similar expression levels to resting KCs and significantly lower levels when compared to blood-MØs (P _1.5 h and 3 h_ < 0.0001). Interestingly, after 5 h of exposure to virulent promastigotes, a sharp decrease on both NOD1 (P < 0.0001) and NOD2 (P < 0.0001) gene expression was observed in blood-MØs (P < 0.0001), evidencing a possible inhibition of immune activation of cell sensors. At this time point, it was observed an inversion on gene expression, with KCs exhibiting higher levels of NOD1 mRNA than blood-MØs (P = 0.0303). Nevertheless, the accumulation of NOD2 mRNA in blood-MØs still was more elevated than in KCs (P = 0.0129). Other evaluated innate immune receptors included cell membrane TLR2 and TLR4, and endocytic TLR9. For these receptors, main variations were registered between blood-MØs and KCs. Similar to what was observed for NOD1 and NOD2, non-stimulated blood-MØs naturally presented higher levels of TLR2 (P _1.5 h_ = 0.0021, P _3 h_ < 0.0001 P _5 h_ = 0.0004) (Figure 4C) and TLR9 mRNA (P _1.5 h_ < 0.0001, P _3 h_ = 0.0057 P _5 h_ < 0.0001) (Figure 4E), when compared to resting KCs. Interestingly, TLR4 gene expression (Figure 4D) were similar between non-stimulated blood-MØs and resting KCs, except at early incubation time (P _1.5 h_ = 0.0172). In addition, TLR2, TLR4 and TLR9 gene expression remain constant in non-stimulated blood-MØs and KCs during the experiments.

Exposure to *L. infantum* promastigotes induced a significant increase of TLR2 (Figure 4C) (P _1.5 h and 3 h_ < 0.0001) and TLR4 (P _1.5 h and 3 h_ < 0.0001) mRNA levels (Figure 4D) in blood-MØs when compared with non-exposed cells. High gene expression of TLR2 and TLR4 may reflect the recognition of *L. infantum* antigens by PRRs and potential blood-MØs activation. KCs exposed to *L. infantum* amastigotes maintained TLRs gene expression similar to resting KCs and exhibit significant lower levels of TLR2 (P _1.5 h_ < 0.0001; P _3 h_ = 0.0019), TLR4 (P _1.5 h and 3 h_ < 0.0001) and TLR9 (P _1.5 h_ = 0.0003; P _3 h_ = 0.0059) when compared to parasite exposed blood-MØs. Interestingly, TLR4 expression in infected blood-MØs showed a peak at 3 h of parasite exposure, being the number of mRNA copies significant different when compared with the previous time-point (1.5 h) (P _1.5 h −3 h_ = 0.0209) (Figure 4D). Regarding TLR9 gene expression in blood-MØs, it was observed a significant decrease in gene expression after exposure to *L. infantum* virulent promastigotes (P _1.5 h_ = 0.0002) (Figure 4E). At 5 h of parasite exposure, blood-MØs exhibited a significant reduction in mRNA levels of all analyzed TLRs: TLR2 (P < 0.0001), TLR4 (P < 0.0001), and TLR9 (P < 0.0001). Remarkably, TLR2 expression in blood-MØs exposed to parasites was lower than non-exposed cells (P _5 h_ < 0.0001). A similar result was obtained for TLR4 (P = 0.0084) and TLR9 (P < 0.0001). KCs exposed to amastigotes did not evidence any marked reduction or increase on analyzed PRRs gene expression, maintaining constant levels, similar to resting KCs, throughout incubation time. Nevertheless, after 5 h of incubation with parasites, TLR2 gene expression was slightly increased compared to non-exposed KC cells and blood-MØs, although not statistically significant. However, TLR9 gene expression in infected KCs was higher compared to infected blood-MØs (P = 0.028). Although, it was observed in infected blood-MØs a sharp reduction on PRRs gene expression after 5 h of parasite exposure, infected cells remain viable, even showing increased lifespan (Figure 3A).

To compare the relative gene expression of analyzed PRRs in promastigote exposed blood-MØs and in amastigotes exposed KCs, heat map representations were generated (Figure 4F,G). Infected blood-MØs exhibited a strong increase in TLR4 and a slight increase in NOD1 expression from 1.5 h to 3 h of exposure to virulent parasites, suggesting that from all the tested PRRs, TLR4 and NOD1 could be engaged in parasite’s antigen recognition. Interestingly, KCs exposed to *L. infantum* amastigotes revealed a different PRR pattern of induction (Figure 4G). Although expressing lower levels of PRRs than infected blood-MØs, parasite exposed KCs showed a strong increase of TLR2 mRNA (after 5 h incubation time) and a moderate rise of NOD1 (3 h to 5 h incubation time) and TLR4 (from 1.5 h to 5 h exposure to parasites) gene expression during the incubation time. Altogether suggesting that TLR2, TLR4, and NOD1 could be involved in parasite antigen’s recognition by KCs.

### 3.4. Blood-MØs Phagocyte Parasites While KCs Release Extracellular Traps

Blood-MØs and KCs were analyzed by scanning electron microscopy (SEM) for cellular morphology alterations resulting from cellular immunological activation (Figure 5). After 3 h exposure to virulent *L. infantum* promastigotes, blood-MØs emitted long and thin pseudopodia to entrap promastigotes, which are compatible with active cellular phagocytosis (Figure 5B,C). Canine KCs, after 5 h of exposure to amastigotes, exhibited morphological alterations compatible with the formation of extracellular traps (ETs), with the complete extrusion of cytoplasmatic cell content and formation of fibers (Figure 5E,F). This constitutes the first evidence of ETs emission by canine KCs activated by *L. infantum* amastigote exposure.

### 3.5. L. infantum Primes Blood-MØs for M2 Phenotype

**Figure 6:**
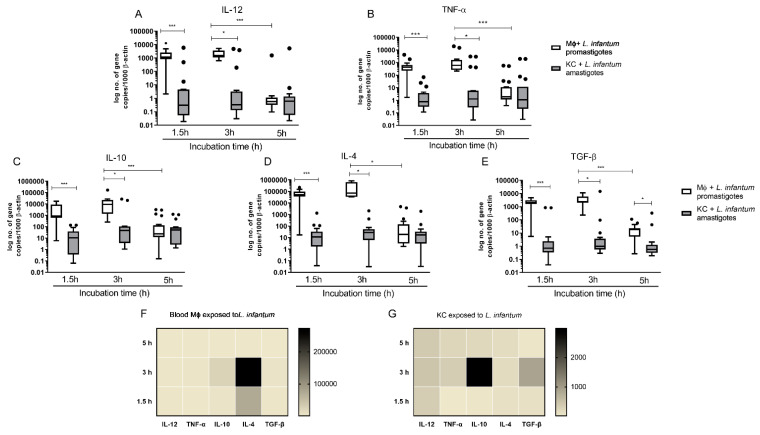
Gene expression of pro-inflammatory and anti-inflammatory cytokines by *L. infantum* infected KCs and blood-MØs. Blood-MØs were exposed to promastigotes and KCs to amastigotes. The number of copies of IL 12 (**A**), TNF-α (**B**), IL 10 (**C**), IL-4 (**D**), and TGF-β (**E**) was determined by real-time PCR at 1.5 h, 3 h, and 5 h. Results are expressed by whiskers box plots (Tukey) (*n* = 10). Outlier values are indicated by black dots. The non-parametric Wilcoxon test was used for statistical comparisons. * (*p* < 0.05) and *** (*p* < 0.0001) indicate significant differences. Heat maps represent the accumulation of pro-inflammatory and anti-inflammatory cytokine mRNA by blood-MØ (**F**) and KCs (**G**) exposed to *L. infantum* parasites.

Alteration in gene expression of PRRs was accompanied by significant variations on key macrophage cytokine in blood-MØs and KCs exposed to virulent *L. infantum* parasites. In Figure 6 a comparison of the cytokine profile generated by the two different macrophage populations after *L. infantum* infection is shown in detail. Overall, blood-MØs exhibited higher levels of gene expression of cytokines than KCs, but only for the first 3 h of parasite exposure. Generation of pro-inflammatory interleukin (IL)-12 and tumor necrosis factor (TNF)-α by blood-MØs exposed to *L. infantum* virulent promastigotes was significantly higher at time points 1.5 h (P _IL-12_ < 0.0001 and P _TNF-α_ < 0.0001) and 3 h (P _IL-12_ = 0.0010 and P _TNF-α_ = 0.0002) when compared with KCs exposed to amastigotes (Figure 6A,B). Interestingly, and reflecting the decrease of accessed PRRs, at 5 h post-parasite exposition cytokine gene expression in blood-MØs significantly decays when compared with the previous time point (3 h) (P _IL-12 and TNF-α_ < 0.0001). KCs maintained lower and constant levels of IL-12 and TNF-α mRNA.

Analysis of gene expression of anti-inflammatory cytokines IL-10, IL-4 and transforming growth factor (TGF) -β revealed that blood-MØs exposed to *L. infantum* promastigotes exhibited higher levels of IL-10 (P _1.5 h_ < 0.0001; P _3 h_ = 0.0411), IL-4 (P _1.5 h_ < 0.0001; P _3 h_ = 0.0313), and TGF-β (P _1.5 h_ < 0.0001; P _3 h_ = 0.0039; P _5 h_ = 0.0240) gene expression when compared to KCs exposed to amastigotes (Figure 6C–E). After 5 h of exposure to parasites, mRNA accumulation of anti-inflammatory cytokines significantly decays when compared with the previous time point (3 h) (P _IL-10 and TGF-β_ < 0.0001; P _IL-4_ = 0.0003). KCs exposed to amastigotes maintained lower and constant levels of IL-10, IL-4 and TGF-β copy numbers when compared with highly inducible blood-MØs.

To compare the relative cytokine generation in *L. infantum* promastigote exposed blood-MØs and in *L. infantum* amastigotes exposed KCs, heat map representations were generated (Figure 6F,G). Infected blood-MØs exhibited a strong increase in IL-4 and IL-10 as dominantly expressed cytokines after 3 h of contact with parasites (Figure 6F). The generation of high levels of IL-4 may be correlated with primming for the M2 macrophage phenotype. Interestingly, KCs exposed to *L. infantum* amastigotes revealed a different cytokine pattern (Figure 6G). Although expressing lower levels of cytokines that infected blood-MØs, after 3 h of parasite exposure KCs strongly generated high levels of IL-10 mRNA and moderate levels of TGF-β. However, parasite-exposed KCs were also able to generate pro-inflammatory IL-12 and TNF-α mRNA during the parasite incubation time.

### 3.6. KCs Are Primed for Tolerance, but Can Generate High Levels of IL-12

**Figure 7:**
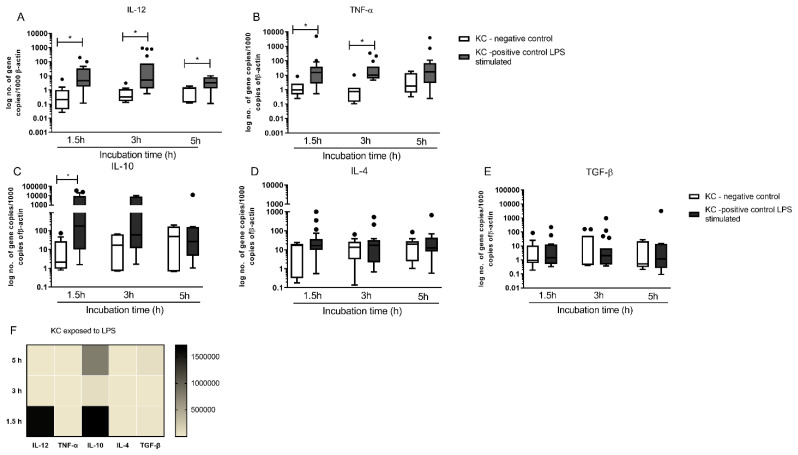
Kupfer cells respond to LPS. Cytokine gene expression of KCs stimulated by LPS for 1.5 h, 3 h and 5 h were evaluated by RT-PCR [IL 12 (**A**), TNF-α (**B**), IL 10 (**C**), IL-4 (**D**), and TGF-β (**E**)] and results expressed by whisker box-plot (Tukey) (*n* = 10). Outlier values are indicated by black dots. The non-parametric Wilcoxon test was used for statistical comparisons. * (*p* < 0.05) indicate significant differences. The relative accumulation of pro-inflammatory and anti-inflammatory cytokine mRNA by LPS stimulated-KCs is represented by heat map (**F**).

To test the natural priming of KCs to immunotolerance, these cells were stimulated by LPS and the cytokine profile was analyzed (Figure 7). KCs were able to react to LPS, significantly increasing IL-12 gene expression over time (P _1.5 h_ = 0.0015; P _3 h_ = 0.0023; P _5 h_ = 0.0307) as well as TNF-α expression (P _1.5 h_ = 0.0006; P _3 h_ = 0.0015) (Figure 7A,B). However, LPS reactive KCs also showed increasing IL-10 gene expression at time point 1.5 h (P = 0.0038), which was maintained until the last time point (Figure 7C). In addition, a slight increase in IL-4 gene expression was observed in LPS stimulated KCs (Figure 7D) although not statistically significant. TGF-β mRNA levels did not reveal any alterations in comparison with non-stimulated KCs.

The heat map representation (Figure 7F) of cytokine gene expression in LPS stimulate KCS highlights an early and transient predomination of IL-12 and IL-10 mRNA high copy numbers over the other evaluated cytokines. Taken together, these results indicate that KCs are viable, reactive to immune stimuli and able to generate pro-inflammatory and anti-inflammatory cytokines for at least five hours of in vitro stimulation.

## 4. Discussion

In the past decades, it has been accentuated the importance of exploring and unravelling MØs, as these cells constitute a complex and pleiotropic population with a wide distribution in the mammal body. MØs undertake several vital functions such as preserving tissue homeostasis, phagocyting and neutralizing pathogens, as well as participating in tissue remodeling during the normal healing process. Understanding the MØ maturation process together with the effect of tissue-microenvironment priming on the behavior of mature MØs has a special interest in the context of infectious diseases. MØs are key cells in several pathophysiology processes, being closely related to the onset of several diseases, including the development of inflammatory disorders, cancer, as well as in CanL, depending on their activation phenotype [7,19]. In CanL, blood-MØs and KCs constitute two different MØs populations with a determinant role in disease outcome.

Mammals have several specialized MØs populations in the body, usually associated with tissues, that differ from blood-MØs linage in source as well as immunological behavior. KCs, constitute the largest tissue-resident MØ population in the mammal body and play a vital role in maintaining the immune homeostasis of the liver. As KCs have a different ontogeny from blood-MØs, it may be of value to consider if the different ontogeny may influence their functional properties. Several studies have shown that different MØs populations exhibit distinct transcriptional signatures [20,21] and epigenetic marks [21,22] that are specific to their tissue of origin, suggesting that the behavior of tissue-specific MØs is not strictly dependent on their ontogeny and that the surrounding microenvironment imprints MØs locally [23]. Thus, it is interesting to notice that even after being isolated from the liver and maintained in vitro, KCs retain the immune tolerance primed by their original tissue location, mainly exhibiting high gene expression of regulatory IL-10 cytokine when exposed to potential threats, such as *L. infantum* amastigotes or bacterial LPS.

Blood-MØs and KCs express several PRRs, as well as phagocytic receptors, such as Fc receptors and the complement receptors [24], molecules of class I and II of major histocompatibility complex (MHCI and MHCII) and co-stimulatory molecules, that enable these cells to process and present antigens to other immune cells, and also become immune activated [25]. Blood-MØs constitute the main host cells of *L. infantum* and is within the circulating blood-MØs that the parasite is transferred into the inner organs, like the spleen and liver. *L. infantum* parasites have long evolved strategies to be able to invade and subvert canine MØs defenses and establish inside the host, reflecting a long parasite-mammal adaptation. It seems that *Leishmania* has evolved by exploiting the natural blood-MØs avidity to phagocyte pathogens. This parasite has developed approaches to promote an efficient uptake by MØs and regulate phagosome maturation, making it more welcoming for parasite growth and preventing parasite destruction. As a result, MØs defenses, such as the generation of oxygen reactive species (ROS), antigen presentation, immune activation, and even apoptosis become compromised, placing MØs in an anergic state, whereas nutrient availability for parasites growth is enhanced [26,27]. As such, in the present study, it was observed that *L. infantum* infected blood-MØs presents higher cellular viability and expanded lifespan, probably induced by the presence of internalized parasites. Many *Leishmania* survival factors are involved in shaping the phagosome and reprogramming MØs to boost infection [28,29]. Recently, particular attention has been given to *Leishmania* ability to manipulate the hypoxia-inducible factor 1 pathway in the host MØs, favoring parasite survival, increasing glucose availability, reducing immunologic reaction, and ultimately prolonging the lifespan of the infected cell [30]. Initial activation of dog blood-MØs towards *L. infantum* is followed by parasite silencing and subversion of MØs defenses, as well as the extended lifespan of infected cells.

Interestingly, KCs reveal a different response pattern from blood-MØs when facing *L. infantum*. Although, being less prompt to activation, since the liver microenvironment favors the immune tolerance [15,31,32], KCs express lower PRRs levels as well as generate reduce levels of cytokine in comparison to blood-MØs. The liver constitutes a main target organ in CanL, but differently from the spleen that stays chronically infected and permits parasite replication, infection in the liver is usually self-containing in asymptomatic animals. These animals, in contrast to symptomatic animals, usually present several well-organized granulomas in the liver, confining the parasites, displaying an effective immunity in an environment of effector T cells. KCs are key cells in this process as they are the starting point for cells recruitment, namely lymphocytes for the control of the infection. In the present work, local immune activation was inferred by the analysis of the PRRs and cytokine profiles generated by cells exposed to *L. infantum*. Both blood-MØs and KCs express several PRRs. The engagement of these receptors leads to the activation of nuclear factor-kappa B which mediates the synthesis of pro-inflammatory cytokines, chemokines, and the production of reactive oxygen and nitrogen species. Therefore, their role in disease appears to be of crucial importance and their role in CanL has been slowly defined over the years. Remarkably, blood-MØs and KCs exhibit different PRRs gene expression when facing *L. infantum* parasites. Infected blood-MØs evidence strong upregulation of membrane-associated TLR4 and cytoplasmatic NOD1 during the first 3 h of infection. Infected KCs, although presenting lower levels of PRRs than infected blood-MØs, upregulate membrane-associated TLR2 and TLR4, and cytoplasmatic NOD1 mRNA. Altogether, these findings suggest that parasite antigens were recognized at the cell’s membrane and also in the cytoplasm after parasite internalization, potentially activating an anti-*Leishmania* immune response. The expression of more than one TLR in the presence of *Leishmania* parasites has been described in *L. infantum* infected dogs, whereas increased expression of TLR2 in the colon was associated with higher parasite load while TLR9 was associated with a lower parasite load in the jejunum [33]. Hosein and colleagues [34] described increased TLR2 transcription and TLR9 downregulation in the whole liver tissue of *L. infantum* experimentally infected dogs. Together these pieces of evidence point towards a very significant role for TLRs in safeguarding against CanL. On the other hand, there is a strong connection between overall TLR downregulation and disease progression. *Leishmania* parasites have created strategies to hijack the innate immune responses [34] in an attempt to survive and complete their life cycle. Interestingly, the role of NODs in CanL is yet to be clarified as previous studies considered its transcription levels in canine monocyte-derived MØs as low and as negligible, after 24 h and 72 h post-*L. infantum* infection [35]. These observations may already reflect the parasite silencing effect observed after 5 h post-infection in the present study for canine blood-MØs. Our findings indicate that blood-MØs can initially recognize *L. infantum* parasites and transiently upregulate NOD1 expression, being subsequently silenced. KCs also activate NOD1 gene expression in the presence of the parasite but later (after 5 h of exposure to *L. infantum* parasites), confirming that cytoplasmatic NOD1, together with other PRRs, may be of major importance in shaping the host immune response during canine *L. infantum* liver infection.

Regarding the generation of cytokines triggered by the parasite presence and internalization by blood-MØs and KCs and consequent PRRs activation, KCs response appears to be more efficient in controlling parasite infection, even with a limited immune activation to avoid excessive inflammation, but sufficient to contribute to the liver natural control of *L. infantum* dissemination. Although KCs primed for immunological tolerance, once infected these cells upregulate anti-inflammatory cytokines. Exposure to *L. infantum* amastigotes lead KCs to generate IL-12 and TNF-α, two key pro-inflammatory cytokines able to trigger immune activation, initiating a cascade of immune cell recruitment from the peripheral blood. The cytokine expression profile was also accompanied by NO production creating an immune milieu suitable for immune activation able to control parasite dissemination. Livers of asymptomatic and naturally *L. chagasi* (syn *L. infantum*) infected dogs have been described as exhibiting well-structured granulomas with a predominance of Th1 CD4^+^ and CD8^+^ T cells, together with dendritic cells, expression of MHCII, and CD11c and CD18 integrins in a cellular microenvironment of immune activation with high levels of interferon-gamma (IFN-γ), IL-12 and TNF-α [36,37,38]. A well-structured granuloma is therefore associated with disease control, although in its structure can harbor parasites and allow its survival, exerting at the same time a tidily monitoring of its multiplication, in a steady state known as concomitant immunity. KCs appear to be determinant in achieving this response and parasite control. On the other hand, blood-MØs seem to be easily induced to phagocyte *L. infantum* parasites, activating an immune response burst associated with parasite early infection (until ≈ 3 h), with PRRs and cytokines upregulation, as well as NO production. *L. infantum* parasites, after being phagocyted and entering the cell, rapidly subvert MØs activation by promoting PRRs downregulation and decreasing NO production, but extended the lifespan of infected cells, as observed in the present study. *L. infantum* infected blood-MØs exhibited IL-4 and IL-10 as dominant cytokines. High levels of IL-4 may be correlated with strong priming for M2 macrophage phenotype, strongly associated with *L. infantum* intracellular survival. The polarization of macrophages into M1 or M2 phenotypes is dependent on the signals provided by the microenvironment. The M2 phenotype is usually described as the alternatively activated macrophage, an anti-inflammatory/regulatory subtype that plays a role in tissue repair and normal resolution of inflammation [39]. M2 macrophage phenotype is associated with arginase-1 activation, leading to urea production as well as IL-10 and TGF-β, promoting parasite intracellular survival. Moreira and colleagues [40] concluded that the predominance of the M2 phenotype in dogs with CanL favoured the replication of *L. infantum* parasites in the skin, spleen and lymph nodes. Interestingly, the authors found that the liver was more resistant to *Leishmania* infection and exhibited a lower proportion of M2 macrophages. In the present study, evidence of KCs activation towards M1 phenotype, exhibiting IL-12 and TNF-α upregulation and NO production were found, therefore supporting the hypothesis that KCs low activation can orchestrate a cascade of lymphocyte recruitment, that can be crucial for the parasite control by the liver. As a consequence of the upregulation of IL-10, a regulatory cytokine, low levels of local inflammation are maintained.

In the current study, it was also observed that KCs react to the presence of *L. infantum* amastigotes by producing extracellular traps (ETs), that can immobilize and kill the parasite. On the other hand, blood-MØs appear to promote promastigote phagocytosis, leading to parasite internalization. Macrophage extracellular traps (METs) are formed by a unique series of cellular events by which cell’s nuclear contents, including chromatin, mix with cytoplasmatic proteins and are extruded from the cell body to form extracellular structures capable of “trapping” and killing microorganisms [41]. Originally first reported to occur in neutrophils, other leukocytes including mast cells, eosinophils, and basophils are now known to produce ETs structures [42,43]. It has also been proposed that METs may act synergistically with other components of host defense. The formation and role of neutrophil extracellular traps (NETs) constitute the most described type of “ETosis”, a form of new cell death pathway that leads to the formation of the ETs [41,42]. In the context of CanL, the formation of NETs has been recently described as part of the natural mechanism of host defense against *Leishmania* parasites, with the ability to significantly reduce parasite viability [12,44,45] and to control infection [46]. Interestingly the role of METs in CanL is not yet fully clarified, in particular for METs formed by KCs, but appear to be part of an essential host mechanism of resistance to the parasite. It is described that *Leishmania* parasites carefully controls the exposition of their antigens subverting MØs capability to process and present antigens and decreasing their capacity to provide optimal TCR signaling to specific effector T cells [47]. Therefore, the formation of METs by KCs enables the exposition of parasite antigens in the liver milieu and may activate local immune responses.

**Figure 8:**
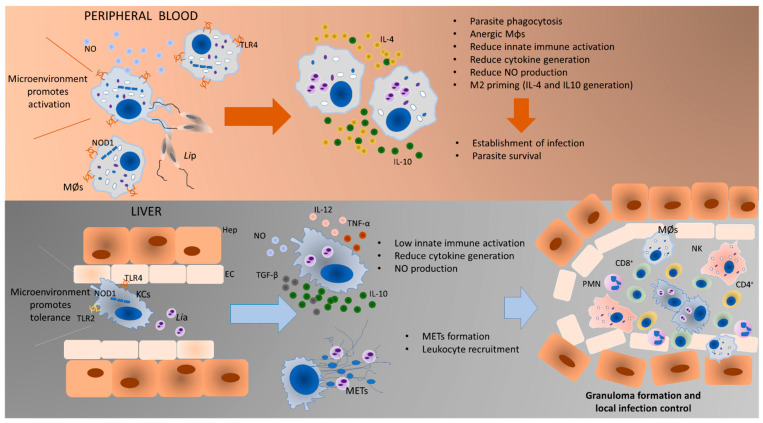
Proposed model of *Leishmania-*MØs interaction in blood and liver. Blood-MØs are rapidly activated in the presence of *L. infantum*, upregulating innate sensors (PRRs) and immune mediators (cytokines). However, shortly after parasite internalization blood-MØs became anergic and permissive to parasite survival. In the liver, KCs are primed by the microenvironment towards immune tolerance and exhibit low immune activation towards *L. infantum*. However, these cells can generate low levels of pro-inflammatory cytokines, release NO and emit METs in the presence of amastigotes, which will recruit other leukocytes and may initiate the constitution of a granuloma. The formation of granuloma is a typical hepatic structure associated with parasite restriction and local control of the infection. MØs-blood macrophages; *Li*p–*L. infantum* promastigotes; KCs-Kupffer cells; *Li*a–*L. infantum* amastigotes; METs-macrophage extracellular traps; TLR4-Toll-like receptor 4; TLR2-Toll-like receptor 2; NO-nitric oxide; NOD1-nucleotide-binding oligomerization domain-like (NOD) receptor 1; Hep-Hepatocyte; EC-Endothelial Cell; CD8^+^-cytotoxic T lymphocytes; CD4^+^-helper T lymphocytes; NK-Natural Killer cells; PMNs-Polymorphonuclear leukocytes.

Based on accumulated experimental pieces of evidence, the authors propose a model of *Leishmania*-MØs interaction for blood-MØs and KCs (Figure 8). Blood-MØs, are “ready for action” cells by nature, with a myriad of immune tools, and are rapidly activated in the presence of *L. infantum*, upregulating TLRs and NODs, particularly TLR4 and NOD1, and release NO. However, shortly after parasite phagocytosis and internalization, blood-MØs became anergic and immune-dormant. These M2 primed cells upregulate anti-inflammatory cytokines (IL-4 and IL-10) and support parasite survival, evidencing a long parasite-host adaptation process, since *L. infantum* can subvert the immune activation state of the blood-MØs. In the liver, KCs are primed by the microenvironment towards immune tolerance and exhibit lower immune activation when encountering *L. infantum* amastigotes. However, KCs can sense the *L. infantum* amastigotes by cell’s innate immune receptors, particularly transmembrane TLR2 and TLR4 and intracellular NOD1, generating low amounts of IL-12 and TNF-α, produce NO, and emit METs, favoring the availability of parasite antigens in the liver milieu which can lead to the recruitment of other leukocytes, especially CD8^+^ T cells and natural killer cells that can initiate the formation of a granuloma response. Granuloma is a typical liver structure associated with parasite restriction and local control of the infection [48]. However, the liver is a complex organ and the orchestration of the anti-*Leishmania* immune response seems derived from a multifaceted interaction between several types of cells. Hepatocytes represent the majority of the liver cells and are normally associated with metabolic functions. Yet, hepatocytes have been recently implicated in the orchestration of the liver’s anti-*Leishmania* immune response, by playing a potentially key role in the crosstalk between liver cells, as hepatocytes are also able to sense and react to the parasite, upregulating PRRs as well as generating immune mediators [32,49], therefore potentially amplifying the immune activation signals emitted by KCs.

## 5. Conclusions

The present work demonstrated the immune activation potential of two different lineages of MØs in the context of CanL and highlighted the close evolution of *Leishmania* and dog macrophage. *L. infantum* can take advantage of the natural predisposition of blood-MØs to be activated and phagocyte pathogens and subvert the cell’s immune activation mechanisms, which allow parasite replication and dissemination, establishing infection in the host and assuring the completion of the parasite life cycle. On the other hand, liver KCs, primed for immune tolerance, are not extensively activated by the presence of this pathogen, allowing *L. infantum* to establish and replicate. However, KCs can activate other mechanisms, such as NO production and METs release, to ensure parasite antigen exposition, launching a cascade of leukocyte recruitment and activation to locally control the infection. Altogether KCs reveal a different response pattern from blood-MØs when facing *L. infantum*. In addition, KCs response appears to be more efficient in controlling this parasite, thus contributing to the ability of the liver to naturally control parasite dissemination. Understanding the mechanism of liver resistance to *L. infantum* infection may lead to a new paradigm in CanL epidemiology regarding the liver as a possible parasite-reservoir organ.

## Data Availability

The data presented in this study are available on request from the corresponding author (G.S.-G.). The data are not publicly available due to confidentiality.

## Supplementary Materials

The following are available online at https://www.mdpi.com/article/10.3390/biology11010100/s1, Figure S1: Flow cytometry immunophenotype of the isolated cells using universal macrophages markers (CD14, CD11c, CD1a) was performed to ensure that the isolated cells were in fact macrophages.
