# Supplementary material for: Zoonotic Visceral Leishmaniasis: New Insights on Innate Immune Response by Blood Macrophages and Liver Kupffer Cells to Leishmania infantum Parasites
Source: Biology (Basel). 2022 Jan 9;11(1):100. doi: 10.3390/biology11010100 (PMC8773027; doi:10.3390/biology11010100)
Supplement: Supplementary file 1 [file biology-11-00100-s001.zip › biology-1511007-supplementary.pdf]

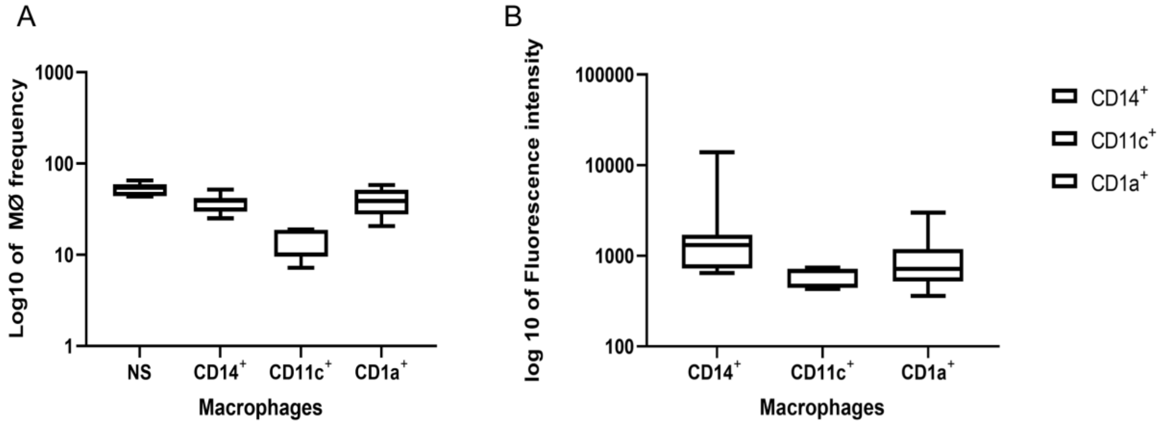


**Figure S1.** Flow cytometry immunophenotype of the isolated cells using universal macrophages markers (CD14, CD11c, CD1a) was performed to ensure that the isolated cells were in fact macrophages.
